# Supplementary material for: Antibiotic-induced decreases in the levels of microbial-derived short-chain fatty acids correlate with increased gastrointestinal colonization of Candida albicans
Source: Sci Rep. 2019 Jun 20;9:8872. doi: 10.1038/s41598-019-45467-7 (PMC6586901; doi:10.1038/s41598-019-45467-7)
Supplement: Supplementary file 1 — Supplementary Materials [file 41598_2019_45467_MOESM1_ESM.docx]

**SUPPLEMENTARY MATERIALS**

**Antibiotic-induced decreases in the levels of microbial-derived short-chain fatty acids correlate with increased gastrointestinal colonization of *Candida albicans***

Jack Guinan^1^, Shaohua Wang^2^, Tony R. Hazbun^3^, Hariom Yadav^2^ and Shankar Thangamani^4^

^1^College of Veterinary Medicine, Midwestern University, 19555 N. 59th Ave. Glendale, AZ 85308, USA.

^2^Department of Internal Medicine-Molecular Medicine, Wake Forest School of Medicine, 575 North Patterson Ave, Winston-Salem, NC 27101, USA.

^35^Bindley Bioscience Center, Purdue University, West Lafayette, IN, USA

^6^Department of Medicinal Chemistry and Molecular Pharmacology, College of Pharmacy, Purdue University, West Lafayette, IN, USA

Bindley Bioscience Center Purdue University, West Lafayette, IN 47906, USA; Department of Medicinal Chemistry and Molecular Pharmacology, College of Pharmacy, Purdue University, West Lafayette, IN 47906, USA.

^4^Department of Pathology and Population Medicine, College of Veterinary Medicine, Midwestern University, 19555 N. 59th Ave. Glendale, AZ 85308, USA.

^5^Bindley Bioscience Center, Purdue University, West Lafayette, IN, USA

^6^Department of Medicinal Chemistry and Molecular Pharmacology, College of Pharmacy, Purdue University, West Lafayette, IN, USA

Correspondence and requests for materials should be addressed to S.T. (email: [sthang@midwestern.edu](mailto:sthang@midwestern.edu))

**MATERIALS AND METHODS**

**Growth assay in pH buffered media**

*C. albicans* strains SC 5314 and ATCC 10231 were grown in RPMI media with or without varying concentrations of acetic, butyric, or propionic acid; RPMI media containing the SCFAs was buffered to pH 7.00 with NaOH. The growth assay was performed as described before ^1^.

**Measuring fungal cell viability using spectrophotometer**

The effect of SCFAs on *C. albicans* strains SC 5314 and ATCC 10231 viability were assessed in the germ tube, hyphae, and biofilm conditions. Briefly, *C. albicans* was seeded at the experiment-specific seeding density and the OD_600 nm_ (initial and later time points) was read for each replicate using a spectrophotometer (OD_600 nm_). The growth of *C. albicans* in each condition was expressed as the % of *C. albicans* growth after the incubation time in each experiment

(OD_600 nm [initial timepoint]_/OD_600 nm [end timepoint]_ X 100).

**SUPPLEMENTARY FIGURES**

**Fig. S1. Effect of SCFAs on *C. albicans* growth in pH-buffered media**

Growth of *C. albicans* strains SC 5314 (a) and ATCC 10231 (b) in pH 7.00 RPMI media or RPMI media containing SCFAs buffered to pH 7.00 with NaOH assessed by spectrophotometer analysis at an optical density of 600 nm after 24 and 48 hours of incubation. Experiment was repeated three times and the three combined replicates were shown here with total n = 9 for each group. Data is represented as means ± SEM. Statistical significance was evaluated using student’s t-test and *P* values (** ≤ 0.01) were considered as significant.

**Fig. S2. Effect of SCFAs on *C. albicans* ATCC 10231 viability under germ tube, hyphae and biofilm conditions.**

*C. albicans* (ATCC 10231) was grown under indicated experimental conditions and fungal cell viability assessed using spectrophotometer analysis. *C. albicans* viability assessed under germ tube (a), hyphae formation and attachment (b), and biofilm (c) conditions. The experiments were repeated three times with n = 9 and combined replicates are shown here. Data is represented as means ± SEM. Statistical significance was evaluated using student’s t-test and *P* values (* ≤ 0.05, ** ≤ 0.01) were considered as significant.

**Fig. S3. SCFAs inhibit *C. albicans* SC5314 hyphae formation *in vitro*.**

*C. albicans* SC 5314 was grown in the presence of SCFAs or in pH-adjusted RPMI media supplemented with 30% FBS and examined using bright field microscopy at 40X (a). Quantification of *C. albicans* hyphae attachment to polystyrene plates in pH-adjusted controls; pH-adjusted controls (12.5 mM and 25 mM) were normalized to the RPMI control (pH 7.00) (b). Quantification of *C. albicans* hyphae attachment to polystyrene plates in SCFA-treatment groups; SCFA treatment groups were normalized to their respective pH controls (c). *C. albicans* viability determined after 12 hours of incubation in hyphae-inducing conditions using spectrophotometer analysis (d). The experiment was repeated three times with n = 18 for the hyphae and n = 9 for the toxicity assays. Combined replicates for both experiments are shown here. Data is represented as means ± SEM. Statistical significance was evaluated using student’s t-test and *P* values (* ≤ 0.05, ** ≤ 0.01) were considered as significant.

**Fig. S4. SCFAs reduce the metabolic activity of fungal cells in the *C. albicans* SC 5314 biofilm.**

*C. albicans* SC 5314 was grown in the presence of SCFAs or in pH-adjusted RPMI and the metabolic activity of the fungal cells in the biofilm was assessed using MTS assay. Percent metabolic activity of fungal cells in the biofilm formed in pH-adjusted controls was determined; pH adjusted controls (12.5 mM and 25 mM) were normalized to the RPMI control (pH 7.00) (a). Percent metabolic activity of fungal cells in the biofilm formed in SCFA-treatment groups; SCFA treatments groups were normalized to their respective pH controls (b). *C. albicans* viability determined after 48 hours of incubation in biofilm-inducing conditions using spectrophotometer analysis (c). All experiments were repeated three times, with n = 18 to determine the metabolic activity in the biofilm and n = 9 for the toxicity assay. Combined replicates for both experiments are shown here. Data is represented as means ± SEM. Statistical significance was evaluated using student’s t-test and *P* values (* ≤ 0.05, ** ≤ 0.01) were considered as significant.

**REFERENCES**

1 Guinan, J., Villa, P. & Thangamani, S. Secondary bile acids inhibit Candida albicans growth and morphogenesis. *Pathogens and disease* **76**, doi:10.1093/femspd/fty038 (2018).
